# Microcollinearity between autopolyploid sugarcane and diploid sorghum genomes

**DOI:** 10.1186/1471-2164-11-261

**Published:** 2010-04-23

**Authors:** Jianping Wang, Bruce Roe, Simone Macmil, Qingyi Yu, Jan E Murray, Haibao Tang, Cuixia Chen, Fares Najar, Graham Wiley, John Bowers, Marie-Anne Van Sluys, Daniel S Rokhsar, Matthew E Hudson, Stephen P Moose, Andrew H Paterson, Ray Ming

**Affiliations:** 1 Department of Plant Biology, University of Illinois at Urbana-Champaign, Urbana, IL 61801, USA; 2 Advanced Center for Genome Technology, Department of Chemistry and Biochemistry, University of Oklahoma, Norman, OK 73019-0370, USA; 3 AgriLife Research Center, Texas A&M University, Weslaco, TX 78596-8344, USA; 4 Plant Genome Mapping Laboratory, University of Georgia, Athens, GA 30602, USA; 5 Department of Botany, University of Sao Paulo, 05508-090, Sao Paulo, SP, Brazil; 6 Energy Biosciences Institute, University of California, Berkeley, CA 94720, USA; 7 Energy Biosciences Institute, University of Illinois at Urbana-Champaign, Urbana, IL 61801, USA

## Abstract

**Background:**

Sugarcane (*Saccharum *spp.) has become an increasingly important crop for its leading role in biofuel production. The high sugar content species *S. officinarum *is an octoploid without known diploid or tetraploid progenitors. Commercial sugarcane cultivars are hybrids between *S. officinarum *and wild species *S. spontaneum *with ploidy at ~12×. The complex autopolyploid sugarcane genome has not been characterized at the DNA sequence level.

**Results:**

The microsynteny between sugarcane and sorghum was assessed by comparing 454 pyrosequences of 20 sugarcane bacterial artificial chromosomes (BACs) with sorghum sequences. These 20 BACs were selected by hybridization of 1961 single copy sorghum overgo probes to the sugarcane BAC library with one sugarcane BAC corresponding to each of the 20 sorghum chromosome arms. The genic regions of the sugarcane BACs shared an average of 95.2% sequence identity with sorghum, and the sorghum genome was used as a template to order sequence contigs covering 78.2% of the 20 BAC sequences. About 53.1% of the sugarcane BAC sequences are aligned with sorghum sequence. The unaligned regions contain non-coding and repetitive sequences. Within the aligned sequences, 209 genes were annotated in sugarcane and 202 in sorghum. Seventeen genes appeared to be sugarcane-specific and all validated by sugarcane ESTs, while 12 appeared sorghum-specific but only one validated by sorghum ESTs. Twelve of the 17 sugarcane-specific genes have no match in the non-redundant protein database in GenBank, perhaps encoding proteins for sugarcane-specific processes. The sorghum orthologous regions appeared to have expanded relative to sugarcane, mostly by the increase of retrotransposons.

**Conclusions:**

The sugarcane and sorghum genomes are mostly collinear in the genic regions, and the sorghum genome can be used as a template for assembling much of the genic DNA of the autopolyploid sugarcane genome. The comparable gene density between sugarcane BACs and corresponding sorghum sequences defied the notion that polyploidy species might have faster pace of gene loss due to the redundancy of multiple alleles at each locus.

## Background

Sugarcane (*Saccharum *spp. L., Poaceae) is a large, perennial, tropical or subtropical grass widely grown primarily for sugar production worldwide. It is a first generation biofuel crop used for ethanol and biomass production as an alternative source of energy [1]. About 75% of the world's sugar (sucrose) supply is from sugarcane and the other 25% from sugar beet (*Beta vulgaris *L., Chenopodiaceae). As a C4 plant, sugarcane has been recognized as one of the world's most efficient crops in converting solar energy into chemical energy, specialized for sucrose production [2-4]. Sugarcane is also among the crops having the most favorable energy input/output ratio [5,6].

The genus *Saccharum *includes six species based on morphology, chromosome numbers, and geographical distribution, and they are *S. spontaneum*, *S. robustum*, *S. officinarum*, *S. barberi*, *S. sinense*, and *S. edule*. Recent genomic and molecular cytogenetic data provided strong evidence that *S. barberi *and *S. sinense *were derived from interspecific hybridization between *S. officinarum *and *S. spontaneum *[7]. Although it has not been proven, *S. edule *is thought to be an intergeneric hybrid between either *S. officinarum *or *S. robustum *with a related genus that might account for its aborted inflorescence [8]. *S. robustum *and *S. spontaneum *are two wild species with different basic chromosomes, x = 10 and x = 8, respectively [9-12]. These two wild species have a wide range of chromosome numbers and ploidy levels with 2n = 60 - 170 for *S. robustum *and 2n = 40 - 128 for *S. spontaneum *[13]. *S. robustum *has been postulated to be the progenitor of the high sugar content species, *S. officinarum *(2n = 8× = 80). The unique basic chromosome number and distinctive DNA fingerprints of *S. spontaneum *from the other species of *Saccharum *are the reasons for a proposal to divide this genus to only two species, *S. spontaneum *as traditionally defined and *S. officinarum *including all other species and interspecific hybrids [13].

Hybrids derived from crosses involving a female *S. officinarum *(2n = 80) and a male *S. spontaneum *exhibit 2n + n transmission, conserving the entire genome of *S. officinarum*, a phenomena known as female restitution [14]. The female restitution remains true in the first backcross between female *S. officinarum *and the 2n + n F_1_, but breaks down at the subsequent backcross. Dutch breeders in early 1900 utilized this unusual phenomenon in sugarcane improvement to integrate resistance genes for biotic and abiotic stresses from the wild species *S. spontaneum *and quickly recover the high sugar content property by a few backcrosses to the high sugar content species *S. officinarum*. For that reason, all current sugarcane cultivars in production are hybrids with 80-90% of the genome from *S. officinarum *and 10-20% of the genome from *S. spontaneum *[15,16].

The complexity of the autopolyploid genome and the interspecific hybridization of modern cultivars hindered progress in genetic/genomic research and the application of genomic tools in sugarcane breeding programs. The only sugarcane bacterial artificial chromosome (BAC) library that we are aware of was constructed from the hybrid cultivar R570 with 2n = 115 chromosomes. Given an estimated genome size of 10 Gb, this BAC library provided 1.3× coverage of the polyploid genome and 14× coverage of the basic chromosome set [17,18]. Sugarcane cultivars used for genetic mapping often have more than 100 chromosomes, and all sugarcane genetic maps constructed to date appear to be incomplete, due to the large number of chromosomes to be mapped and the limited genomic sequences available for developing markers [16,19-22]. This deficiency has restricted the application of marker-assisted selection, because much of the genome cannot yet be scanned for target traits. However, sugarcane is in the forefront as a source of biofuel, and this has stimulated investment from both private and public sectors in sugarcane research. Coupled with the decreased cost of DNA sequencing using the next generation sequencing technologies, the once daunting and prohibitive task of sequencing the autopolyploid sugarcane genome becomes a real possibility.

Sorghum (*Sorghum bicolor *(L.) Moench) is a major cereal crop that provides food, feed, fiber, and fuel. It is domesticated in northern Africa with the ability to be productive in adverse environment and is valued for its drought tolerance. In comparison to polyploid sugarcane, sorghum is a diploid with 10 chromosomes and has a genome of about 730 Mb [23]. The size of the monoploid sugarcane genome is estimated to be of similar magnitude, approximately 930 Mb; the genome complexity in sugarcane comes from the ploidy level and the two genome sets presented in sugarcane hybrids. The sorghum genome has been sequenced because of its small genome size and its importance for food security and biofuel production in diverse environments, particularly developing countries in the tropics [23]. Sorghum and sugarcane belong to the same subtribe, Saccharinae, within the grass family Poaceae [24] and are close relatives to each other, sharing a common ancestor about 8-9 million years ago [25]. The completion of the sorghum genome sequence offered unprecedented opportunities for sugarcane genomic research [23].

The synteny between sugarcane and sorghum genome has been reported before using DNA markers [19], but no details of microsynteny are available except one pair of sugarcane bacterial artificial chromosomes (BACs) containing the *Adh1 *gene [25]. The objectives of this project are: (1) to test the suitability of using the sorghum genome as a template to assist assembly of sugarcane sequences generated from 20 selected sugarcane BACs using 454 Flex; (2) to explore the sequence features of the autopolyploid by examining a large set of long contiguous genomic sequences; and (3) to test the hypothesis of microcollinearity between sorghum and sugarcane at 20 chromosomal locations across the respective genomes.

## Results

### Selection and sequencing of 20 sugarcane BACs

**Figure 1:**
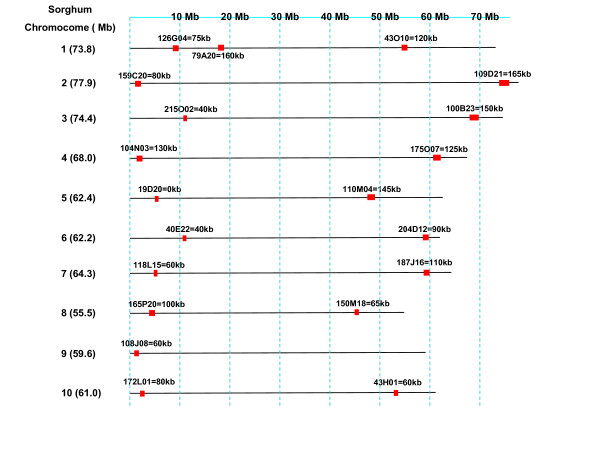
**Orthologous chromosomal locations of selected 20 sugarcane BACs on each chromosome arm of the 10 sorghum chromosomes**. The solid line represents the sorghum chromosome. The red rectangle represents the sugarcane BACs. The locations of the BACs were based on the overgo probe hits on the sorghum scafold sequence. The BAC 79A20 was corresponding to an arm of the sorghum chromosome 9 in an earlier version of the assembly when we started the work and it, and it was on chromosome 1 in the final version of the sorghum genome assembly.

To examine the microsynteny between the sugarcane and sorghum genomes, 20 sugarcane BACs of the hybrid cultivar R570 were selected based on overgo hybridization data performed on the sorghum and sugarcane BAC libraries and locations of the overgo probes in the sorghum sequences [17,26]. Specifically, 3145 overgo sequences were blasted against the sorghum genome assembly (preliminary #7, the basis for the initial published assembly), and 1961 have exactly one hit to the sorghum assembly with at least 35 to 40 bp identical to the sorghum genome target. The blast hit locations were converted to the locations in base pair on the ordered sorghum scaffolds and then to the positions of assembled chromosomes. The 1961 "single copy sorghum overgoes" were compared to the sugarcane hybridization data and 1003 overgoes had 4 to 20 hits on sugarcane as 10 to 12 homologs per basic chromosome were expected in the hybrid cultivar R570. We then found a subset of BACs hit by at least two of the above probes that were within about 50 kb in sorghum and 20 sugarcane BACs were then selected with one sugarcane BAC corresponding to each sorghum chromosome arm in the euchromatic regions of the 10 sorghum chromosomes (Figure 1 and Additional File 1).

These 20 BACs were sequenced by one 454 Flex run using a BAC pooling strategy with four horizontal and five vertical pools (Additional File 2). A total of 593,265 reads were generated, yielding 118 Mb raw sequences after excluding low quality sequences. The insert sizes of the 19 sugarcane BACs with insert were estimated using pulse field gel electrophoresis, ranging from 40 - 165 kb with an average of 97.6 Kb (Table 1). The BAC clone 19D20 turned out to be empty as confirmed by the end sequences of this clone matching 100% to the pBeloBAC11 vector sequence. This clone had been manually selected because the two hybridized overgoes hit same location on sorghum genome, which weaken the anchor evidence of this clone. We then excluded it from further analysis. The total reads for remaining 19 BACs provided 64× coverage for the combined 1.86 Mb BAC sequences.

**Table 1:** Summary of sequencing results of the 20 sugarcane BACs using 454 Flex and the 2 BACs using Sanger method.

General BAC clone information					BAC sequence assembly					Sugarcane - sorghum sequence alignment			
**No**.	**BAC ID**	**Insert size (kb)**	**Chr**^a^.	**GenBank ID**	**No. of contigs**	**Assembled BAC (bp)**	**No. of contig >10 k**	**No. of un-ambigious contigs^b^**	**Total (kb)**	**No. of ordered contigs**	**Total length of ordered contigs (kb)**	**Spanned in sugarcane (bp)**	**Spanned in sorghum (bp)**

1	SC0126G04	75	1	FJ348725	8	86,848	4	7	85	5	79	59,458	61,142

2	SC0043O10	120	1	FJ348717	12	140,528	6	7	119	5	118	92,727	86,118

3	SC0159C20	80	2	FJ348726	22	136,575	4	19	135	8	100	57,277	41,718

4	SC0109D21	165	2	FJ348722	15	323,526	5	9	131	3	93	64,904	59,816

5	SC0215O02	40	3	FJ348732	14	90,423	4	10	79	3	38	26,478	25,458

6	SC0100B23	150	3	FJ348719	21	175,925	4	14	147	7	118	70,340	72,844

7	SC0104N03	130	4	FJ348720	15	131,592	6	13	131	6	95	57,524	50,074

8	SC0175O07	125	4	FJ348729	42	99,784	0	37	92	0	0	\	\

9	SC0019D20	0	5		0	0	0	0	0	0	0	\	\

10	SC0110 M04	145	5	FJ348723	15	147,646	6	11	127	4	99	41,367	37,981

11	SC0040E22	40	6	FJ348716	21	59,035	1	14	47	3	37	23,505	21,900

12	SC0204D12	90	6	FJ348731	5	96,221	3	4	96	2	76	39,070	27,519

13	SC0118L15	60	7	FJ348724	12	101,744	1	8	100	4	83	76,967	81,213

14	SC0187J16	110	7	FJ348730	11	119,506	6	10	119	4	55	31,930	27,526

15	SC0165P20	100	8	FJ348727	12	94,102	1	8	87	1	44	32,797	87,512

16	SC0150 M18	65	8	FJ348733	6	130,463	2	5	129	2	128	113,899	315,724

17	SC0108J08	60	9	FJ348721	12	113,932	3	8	104	5	98	79,663	69,498

18	SC0079A20	160	1	FJ348718	14	199,955	6	7	89	4	77	61,829	65,536

19	SC0172L01	80	10	FJ348728	19	52,373	0	18	50	0	0	\	\

20	SC0043H01	60	10	FJ348715	11	309,732	6	9	122	6	113	56,110	58,070

Total		1855			287	2,609,910	74	218	1989	72	1451	985,845	1,189,649

1	SC0118L15*	60	7	GU207345	6	62,255	3	6	62	6	62	61,625	91,428

2	SC0172L01*	80	10	GU207346	3	82,136	2	3	82	3	82	\	\

*: Sanger sequence; a: corresponding sorghum chromosome; \: not available; a: chromosome number of sorghum based on the overgo probes' location.

b: All 287 contigs were BLAST against each other, and those contigs with matching sequences to other contigs were removed from comparative analysis with sorghum genome

Initial assembly of BAC sequences based on the row and column pools resulted in a total of 287 contigs, ranging from 5 to 42 contigs per BAC with a combined length of 2.61 Mb (Table 1). These assembled contigs were examined to eliminate redundant small assemblies, and 218 unambiguous contigs with a total of 1.99 Mb sequences were sorted out to represent the sequence of 19 BACs with an estimated sum of 1.86 Mb (Table 1). To order the multiple contigs for each BAC, orthologous sorghum sequences were used as templates to orient these sugarcane contigs and fill the gaps between the contigs when possible. A sum of 1.45 Mb sugarcane contigs were unambiguously ordered, accounting for 78.2% of the 1.86 Mb BAC sequences (Table 1). Contigs of two BACs, SC0172L01 and SC0175O07, were not ordered because all the contigs were less than 9 kb with a few genes predicted but projected to different sorghum super contigs. Among the 1.45 Mb ordered sugarcane contig sequences, the sequence aligned with sorghum sequence spanned 0.99 Mb (Table 1), accounting for about 53.1% of the sugarcane BAC sequences.

### Gene content and repetitive sequences

To annotate the sequenced sugarcane BACs, the repetitive sequences of the assembled BAC contigs were first masked by RepeatMasker using a repeat database combined from RepBase databases and TIGR Plant Repeat Databases. The remaining sequences were annotated using sugarcane ESTs, sorghum ESTs, and predicted sorghum gene models. In total, 209 protein coding genes were predicted in the 19 sugarcane BAC sequences, including 155 (74.2%) validated by sugarcane ESTs, 28 by sorghum ESTs, and additional 26 corresponding to sorghum predicted gene models (Additional File 3). In the corresponding sorghum regions, 202 genes were annotated, including 122 (60.4%) validated by sorghum ESTs, 31 by sugarcane ESTs, and additional 49 by sorghum gene models. A total of 171 (81.8%) genes from the 19 BACs are predicted to be true orthologs between sugarcane and sorghum. Sequence identity between these two species across 140 kb coding region revealed a 95.2% exon sequence identity with a range of 80.1 to 100%.

**Figure 2:**
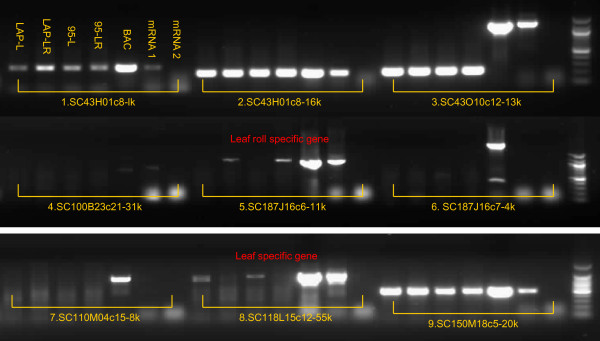
**Images of RT-PCR for testing gene expression of 9 annotated sugarcane genes inferred from sorghum ESTs and predicted genes**. Seven samples were used for RT-PCR amplification, and they are 1. *S. officinarum *LA Purple mature leaf cDNA (LAP-L); 2. LA Purple leaf roll cDNA (LAP-LR); 3. An F1 individual 95-4655 mature leaf cDNA (95-L); 4. 95-4655-leaf roll cDNA(95-LR); 5. BAC DNA as positive control (BAC); 6. Total RNA without DNase treatment from both leaf and leaf roll of LAP; 7. Total RNA with DNase treatment from both leaf and leaf roll of LAP. Genes 1, 2, 3, 5, 8, and 9 are expressed with Gene 5 being leaf roll specific and Gene 8 mature leaf specific. No gene expression were detected in Genes 4,6 and 7.

For the genes of sugarcane inferred by sorghum ESTs and gene models, RT-PCR was used to confirm their expression. Among the 28 genes matched by sorghum ESTs, primers were designed from 26 of them, and 19 (73.1%) were expressed in mature leaves or leaf rolls (young leaves). Among the 26 genes predicted by the sorghum gene models, primers were designed from 21 of them, and 9 (42.9%) were expressed in mature leaves or leaf rolls (Figure 2, Additional Files 4 and 5). The expression of two genes appear to be developmental stage specific, SC187J16c6-11k only detected in leaf rolls and SC11815c12-55k in mature leaf. The number of validated genes on the 19 BACs is 183 (90.6%), and the average gene density is one gene/10.1 kb in these euchromatic regions.

Among these annotated genes, 19 sugarcane genes have no counterpart on the corresponding sorghum region while 12 sorghum genes have no counterpart on the corresponding sugarcane regions. The 19 putative sugarcane-specific genes are distributed on 11 BACs and are all supported by sugarcane ESTs. Among them, 17 have no orthologs in the sorghum genome and 2 have orthologs in other parts of the sorghum genome rather than in the corresponding regions. For the 12 sorghum genes without sugarcane orthologs, one is supported by sorghum EST, and the other 11 are predicted by sorghum gene models. The 17 sugarcane-specific genes were blasted against the non-redundant protein database in GenBank. Only five of them presented significant match (e value < E-06), two with known function, encoding beta-galactosidase 6 and auxin efflux carrier, respectively, and the other three encoding hypothetical proteins. The remaining 12 ESTs have no match in the non-redundant protein database.

We examined the content of repetitive sequences in these euchromatic regions of sugarcane and sorghum. The sorghum genome contains 61% repetitive sequences, but most of them are in the centromeric and pericentromeric regions [23]. The percentage of repetitive sequences in the about 2 Mb regions studied is 25.5% in sugarcane and 27.6% in sorghum. Both genomes contain similar quantities of retroelements, 16.9% in sugarcane and 16.5% in sorghum, mostly LTR-retrotransposons. However, sugarcane has more Copia than Gypsy elements (3.5% vs. 1.2%) while sorghum has more Gypsy (4.8% vs. 1.2%) elements in these analyzed regions. Sorghum displayed more DNA transposons than sugarcane (8.9% vs. 5.9%) in these regions (Table 2).

**Table 2:** Summary of repetitive sequences in sugarcane BACs and the orthologous euchromatic regions in sorghum.

Repeat elements	Sugarcane BAC sequences (1,989,325 bp)		Sorghum homologous region (2,006,815 bp)	
	
	Length (bp)	Percentage of the sequence (%)	Length (bp)	Percentage of the sequence (%)
**Retroelements**	336312	**16.91**	334117	**16.47**

SINEs:	2058	0.1	2647	0.13

LINEs:	14912	0.75	7700	0.38

L1/CIN4	14912	0.75	7700	0.38

LTR elements:	319342	16.05	323770	15.96

Ty1/Copia	70236	3.53	24017	1.18

Gypsy/DIRS1	23381	1.18	97819	4.82

**DNA transposons**	116731	**5.87**	179859	**8.86**

hobo-Activator	3563	0.18	4117	0.2

Tc1-IS630-Pogo	4608	0.23	5595	0.28

En-Spm	23927	1.2	39516	1.95

MuDR-IS905	5529	0.28	4656	0.23

Tourist/Harbinger	44991	2.26	79754	3.93

Unclassified:	36992	1.86	24513	1.21

Simple repeats:	4940	0.25	9719	0.48

Low complexity:	8461	0.43	10488	0.52

**Total**	507807	**25.53**	559303	**27.57**

In the approximately 2 Mb sugarcane sequences, about 5 kb were simple repeats. We have designed 44 pairs of primer from the sequences flanking the simple sequence repeats (SSRs) for genetics mapping. Thirty six SSRs were amplified successfully and six were polymorphic between *S. officinarum *LA Purple and *S. robustum *Molokai 5829, the parents of our sugarcane mapping population (Additional File 6).

### Comparative analysis of sugarcane and sorghum homologous sequences

**Figure 3:**
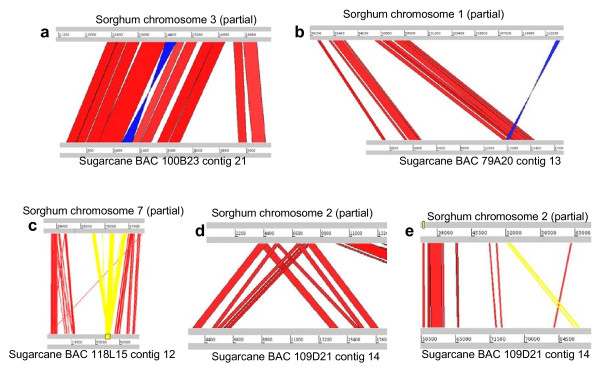
**Rrearrangements in the aligned regions between sugarcane and sorghum genomes**. **a**, inversion; **b**, inverted duplication on sorghum genome; **c**, duplication on sorghum genome; **d**, duplication on sugarcane genome; and **e**, translocation.

The selected sugarcane BACs corresponding to euchromatic regions of sorghum chromosome arms made it possible to analyze microsynteny between these two closely related genomes. Of the 209 genes annotated in the sugarcane BACs, 178 (85.2%) matched orthologous sorghum genes, and they provided the anchoring points for aligning sugarcane BAC contigs to sorghum chromosomes. The sequence of BAC SC79A20 was actually aligned with sorghum chromosome 1 instead of chromosome 9 as defined by previous overgo probes (Table 1), which is due to the reassignment of few scaffolds in the final chromosome assembly. All other BACs aligned correctly to the sorghum chromosome arms as assigned by the locations of overgo probes. Most of the aligned regions are collinear between sugarcane and sorghum, while numerous small scale chromosomal rearrangements were uncovered, including all known types of chromosomal rearrangements (Figure 3 and Table 3). Deletions and insertions are too numerous to be counted. Duplications appeared to have occurred more frequently in sugarcane (26 events) than in sorghum (14 events). Three inversions were detected, but we lacked an outgroup to enable inference about which genome they had occurred in. Translocations occurred more frequently than inversions, with seven translocations and four inverted translocations found.

**Table 3:** Chromosomal rearrangements in the aligned regions between sugarcane and sorghum genomic sequences.

Chromosomal rearrangements	Total length (bp)	Number of events	Average length (bp) per event
Translocation	1,925	7	275

Inverted translocation	1,566	4	392

Inversion	842	3	281

Duplication in sugarcane	12,150	26	467

Duplication in sorghum	4,595	14	328

Total	21,078	54	390

**Figure 4:**
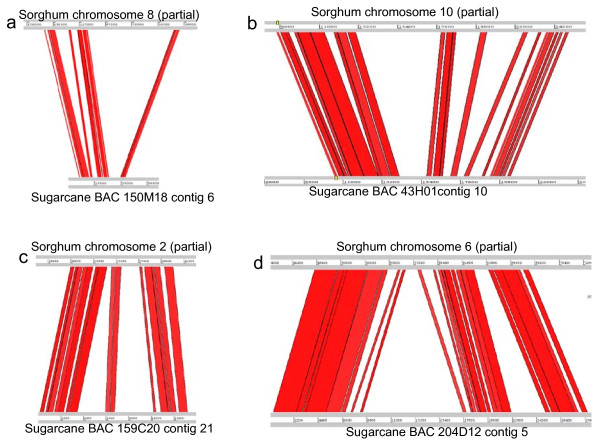
**Genomic sequence expansion in sugarcane and sorghum**. The alignments were performed using a public online program, WebACT. Bottom line represents the sugarcane sequence and top line represents the corresponding sorghum sequence. **a**. The sorghum sequence is expanded 192.7%. **b**. The sorghum sequence is expanded 66.5%. **c**. The sugarcane sequence is expanded 44.1%. **d**. The sugarcane sequence is expanded 47.0%.

**Figure 5:**
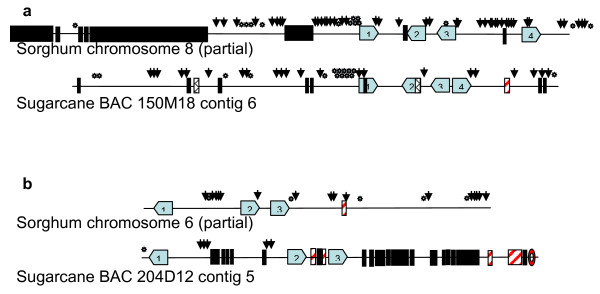
**Organization of two homeologous regions between sorghum and sugarcane**. Genes are indicated by blue boxes; LTR retroelements by black boxes; SINE retroelements by stripped boxes; LINE retroelements by squared boxes; DNA transposons by arrows; simple repeats by stars; centromeric repeats by oval. **a**. Comparison between partial sequence of sorghum chromosome 8 and sugarcane BAC 150 M18 contig 6 (showing expanded sorghum sequence). Proteins giving highest BLAST hit of gene1 to 4 are SEU3A protein; thioredoxin M-type, chloroplast precursor; expressed protein; exonuclease family protein, respectively. **b**. Comparison between partial sequence of sorghum chromosome 6 and sugarcane BAC 204D12 contig 5 (showing expanded sugarcane sequence). Proteins giving highest BLAST hit of the gene1 to 3 are sugar transport protein; hypothetical protein OJ1065_B06.22; and expressed protein, respectively.

Alignment of sorghum and sugarcane genomic sequences revealed local DNA sequence expansion in both genomes (Figure 4). But over all, the sorghum genome expanded more in the aligned regions. Among the sequenced sugarcane BACs, a total region of 986 kb aligned to 1,189 kb sorghum sequence, suggesting a net 204 kb (20.7%) expansion in sorghum, likely due to the accumulation of retrotransposons. To determine the possible cause of sequence expansion, we examined two of the aligned regions where the sorghum sequence expanded 192.7% (Figures 4a and 5a) and sugarcane sequence expanded 47% (Figures 4d and 5b). The genic regions between the two species were highly conserved by presenting a set of orthologous genes with the same size, orientation, structure, and function. However, the intergenic regions were extensively dissimilar with abundant transposable elements, including three distinct retrotransposon, DNA transposons, simple repeats, and centromeric repeats (Figures 5 and Additional File 7), which accounted for the expansion in both sorghum and sugarcane sequences.

Tandem gene duplication was observed in both sugarcane and sorghum genomes. These genes were identified by aligning genome sequence with sugarcane and sorghum ESTs. For example, a gene duplication was found on sorghum chromosome 7 but not in the corresponding region of sugarcane BAC 118L15 contig 12. Three tandem copies of the gene predicted to encode the 60S ribosomal L10A protein is found in sorghum and only one copy in sugarcane. Another gene encoding serine carboxypeptidase 2 was duplicated in sugarcane BAC 109D21 contig 11-13-14 but not in the corresponding region of sorghum chromosome 2. There were two copies of this gene in sugarcane and one in sorghum [23]. In another case, a gene encoding for receptor kinase was duplicated in sugarcane BAC 108J08 but not in the corresponding region of sorghum chromosome 9 (Additional File 8).

The large number of orthologous genes between sugarcane and sorghum allowed us to estimate the divergence time between these two closely related genera to be about 7.7 million years using 67 pairs of orthologous genes (Table 4).

**Table 4:** Estimated divergence time among sugarcane, sorghum, and rice.

	Median Ks	Orthologous Gene Pairs	Divergence Time
Sugarcane-Sorghum	0.10	67	7.7 MYa

Sugarcane-Rice	0.53	50	40.8 MYa

Sorghum-Rice	0.58	49	44.6 MYa

### Validation of 454 sequence assembly using Sanger sequences

To assess the quality of 454 Flex sequence assembly, we selected two BACs SC172L01 and SC118L15 for Sanger sequencing. SC118L15 appeared to harbor a substantial amount of rearrangement between sugarcane and sorghum and SC172L01 had 19 relatively short contigs assembled from 454 reads with the longest contig of 7 kb and a few genes being annotated that scattered in different regions of the sorghum genome. The Sanger sequence reads of these two BACs, were assembled into 3 and 6 contigs, respectively, after multiple attempts to close the remaining gaps by primer walking, each showing a reduction from the 19 and 12 contigs of 454 sequences. The Sanger sequences of these two BACs also matched the estimated insert size of 80 kb for SC172L01 and 60 kb for SC118L15, while the assembled 454 contigs are 52.4 kb and 101.7 kb for these two BACs respectively (Table 1).

**Figure 6:**
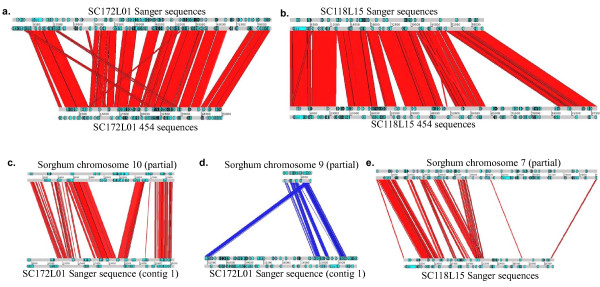
**The sequence alignments between the Sanger sequence of the two BACs with their 454 sequences and orthologous sorghum sequences**. a. Comparison of Sanger sequence and 454 sequence of BAC SC172L01. b. Comparison of Sanger sequence and 454 sequence of BAC SC118L15. c. Alignment of SC172L01Sanger sequence and orthologous sorghum sequence of chromosome 10. d. alignment of SC172L01Sanger sequences with orthologous sorghum sequence of chromosome 9. e. Alignment of SC118L15 Sanger sequences with orthologous sorghum sequence of chromosome 7.

Alignment between the Sanger and 454 sequences revealed missing sequences in each of the assemblies. Segments of SC172L01 are not in the 454 contigs, whereas all assembled Sanger sequences of SC118L15 are in 454 contigs that also have additional sequences not present in the Sanger sequences (Figure 6). Plots of the Sanger and 454 sequences against the sorghum sequences also showed that the 454 sequence of SC118L15 is more complete than the Sanger sequence of this BAC, possibly because of the sequences in the five gaps that we were not able to close (Additional File 9). The Sanger sequence of SC172L01 is more complete than the 454 contigs, which covered only 52.3 kb of the 82 kb insert (Figures 6a and Additional File 9), and allowed us to identify and annotate an extra gene and a few more retrotransposase genes validated by the sugarcane ESTs. These two sugarcane BACs contains 41.8% repetitive sequences, higher than the 29.0% in the sorghum homologous regions (Additional File 10).

The previous 454 sequence of SC172L01 had 19 short contigs (each less than 8 kb) and six genes including four retrotransposase genes. Its alignment to sorghum sequence was puzzling because the six genes aligned to different regions of the sorghum genome. The more complete Sanger sequences of SC172L01 allowed us to align it with the sorghum genome, which revealed chromosomal rearrangements after the divergence of these two species. The BAC SC172L01 aligned largely to sorghum chromosome 10, but a portion aligned to chromosomes 8 and 9 (Figures 6c, d, and Additional File 9). Though the Sanger sequence of SC118L15 was less complete than its 454 sequence, the assembly from Sanger reads further confirmed the local chromosome rearrangements harbored by this BAC (Figure 6e).

## Discussion

Because of the large number of chromosomes (often >100) and the nature of autopolyploids, both high density genetic mapping and physical mapping have proven to be challenging tasks in sugarcane. Currently, there is no physical map and no saturated genetic map that covers all chromosomes. Alternative approaches would need to be tested for a potential sugarcane genome sequencing project. Our results showed that the sorghum genome is an excellent template for assembly of sugarcane euchromatic sequences. The initial assembly of pooled 454 BAC sequences showed 40% inflation compared with estimated insert sizes, which likely was caused by multiple assemblies of repetitive sequences. After aligning the sequences with the sorghum genome using orthologous genes as anchors, 78.2% of the sugarcane BAC contigs could be ordered unambiguously and 53.1% of the sugarcane BAC sequences aligned with the sorghum genic regions. Sequences that were not aligned consisted of repetitive and non-coding sequences.

The suitability of the sorghum genome as a template for sugarcane genomic sequence assembly, at least for the genic regions, will be critical for strategic planning to sequence the sugarcane genome. Current BAC by BAC or whole genome shotgun sequencing approach would require a high density genetic map ideally with a density at two markers per Mb and a physical map with a10× genome coverage. For sugarcane, the only BAC library available is constructed from commercial hybrid cultivar R570 with 1.3× genome coverage [17]. A 10× coverage BAC library would require one million clones with an average insert size of 100 kb, an expensive and laborious task. A high density genetic map would require mapping 20,000 markers for the 115 chromosomes of R570, and these markers would have to be sequence tagged to be useful for sequence assembly, not anonymous markers such as amplified fragment length polymorphism (AFLP) markers. In the past 20 years, 13 sugarcane maps have been constructed, and each of them covers only a fraction of the genome with less than 2,000 markers, and the majority of the markers in recent maps are AFLP markers [16,19-22]. Fortunately, the cost of sequencing is declining rapidly with increased throughput. Most likely, a draft of the sugarcane genome will be generated before an ultra high density (2 markers per Mb) genetic map and a physical map are available, using the sorghum genome as a template for sequence assembly.

The sugarcane genome has gone through at least two rounds of genome wide duplication events to become an octoploid since its divergence from a common ancestor shared with sorghum. The two rounds of duplications might have occurred after the speciation event separated the two wild species *S. robustum *(x = 10) and *S. spontaneum *(x = 8) since these two species has different basic chromosome number [9-12], within 2 million years [25]. Although each octoploid has eight genomes, it is not possible to distinguish each individual genome and every genome is a mosaic of all eight genome segments, because every chromosome is free to pair and recombine with any one of the other seven homologous chromosomes during meiosis, although it should be noted that most genetic maps of sugarcane showed some evidence of preferential pairing [19,27]. For this reason, it might not be possible to distinguish the two recent genome wide duplications, and a minimum tiling path of BAC clones would be as a good representative as any one single genome in the octoploid. The hybrid cultivar R570 has 2n = 115 chromosomes with potentially 12 genomes. We selected a single BAC from each of 20 euchromatic regions corresponding to 20 distinctive chromosome arms (ended up with 18 arms due to the empty clone and a misplaced BAC), representing one of the potential 12 genome. We found more genes in sugarcane sequenced fragments than in sorghum in the aligned homologous regions (209 vs. 202), and more putative sugarcane specific genes (17) than sorghum specific genes (12). Two of the 19 initially annotated sugarcane specific genes have orthologs in other part of the sorghum genome, which left 17 to be most likely sugarcane specific genes. All 17 putative sugarcane-specific genes were validated by sugarcane ESTs, while only one of the 12 putative sorghum-specific genes was validated by sorghum ESTs. Moreover, 12 of the 17 sugarcane specific genes have no match in the non-redundant protein database in GenBank, suggesting that they are likely involved in sugarcane-specific processes. Although we masked the repetitive sequences of the BACs using plant repeat database, it is possible that some of them could be low copy transposable elements since we don't have a sugarcane specific repeat database.

The sugarcane EST project (SUCEST) yielded a database containing 237,954 ESTs assembled into 33,620 unigenes from 26 different cDNA libraries [28]. This EST database validated 74.2% of the 209 annotated genes on the 19 sugarcane BACs, while only 60.4% of the 202 sorghum annotated genes were validated by sorghum ESTs. It might be a general rule that the EST databases of polyploid organisms represent higher percentage of genes than their diploid counterparts, because the multiple (12 in the case of sugarcane hybrids) allelic forms of each gene would result in greater chance of a particular allelic form to be sequenced in a collection of a wide range of tissues and developmental stages. However, more alleles don't necessarily increase the chance of a particular gene to be expressed in any type of tissues or developmental stages, as we have discovered two developmental stage specific genes in our RT PCR experiment involving 47 predicted genes.

The subtribe Saccharinae includes three major biofuel crops, sugarcane, *Miscanthus*, and sorghum. Sugarcane and *Miscanthus *are closely related and belong to the *Saccharum *complex [8]. Sorghum is their closest relative outside of the *Saccharum *complex. Our estimate of a common ancestor shared by sugarcane and sorghum about 7.7 million years ago is in line with the 8-9 million years estimated by Jannoo et al [25]. This time frame should be also applied to *Miscanthus *as it is a member of the *Saccharum *complex.

Most of the BAC sequences aligned with the sorghum sequences collinearly. However, one of the BAC (172L01) aligned to multiple chromosomes of sorghum, indicating large scale chromosomal rearrangements between sugarcane and sorghum genomes. Numerous local small scale (within a BAC) rearrangements between sugarcane and sorghum genomes were also detected. These sequence arrangements at both intra- and inter-chromosomal scales between the two species reflect their evolutionary history after their divergence about 8 million years ago. Our sugarcane BAC sequences provide the view of a representative genome of the possible 12 genomes in R570. It would be more interesting to document the rearrangements among sugarcane homologs, which should be far fewer.

The 2C genome size of R570 is about 10 Gb with an average of 87 Mb per chromosome among its 115 chromosomes, larger than the ~73 Mb per chromosome in sorghum [23]. However, our data suggest that the sorghum sequences appear to be expanded compare to the sugarcane orthologous sequences studied, due to accumulation of retroelements, contradicting the genome size estimates from flow cytometry. If what we observed truly reflect the features of these two genomes, the basic genome of sugarcane (x = 10 or x = 8) could be smaller than that of sorghum. The discrepancy between the direct sequence comparison and the genome size estimates could be due to tendency of overestimating genome size by flow cytometry, as demonstrated by the sequenced genomes of rice and poplar [29,30]. It is also possible that the discrepancy is caused by inaccurate assembly of repetitive sequences of the sugarcane BACs generated by 454 Flex. Finally, the small sampling of sugarcane BACs that we studied may not be representative of the genome as a whole.

Sugarcane has been cultivated and improved over thousands of years, beginning in prehistoric times with selection initially on natural variations and continuing with the modern techniques of hybridization and genetic engineering. Enormous yield increase has been achieved in the last century by breeding for yield, disease and insect resistance, and stress tolerance. While sugarcane farmers throughout the world face constant challenges to sustain profitability and protect the environment [31], breeders face not only those challenges but also a biological constraint as the gap between average farm yield and genetic yield potential is narrowed through improved agronomic practices [32]. Sequencing the complex genome of autopolyploid sugarcane will provide the genomic resources to study genes and gene interactions controlling sugar yield, biomass yield, and other agronomic traits. A sugarcane genome sequence has the potential to revolutionize sugarcane improvement programs by providing high throughput genome wide screening for genomic selection [33], and for mining promoters of specific alleles.

## Conclusion

Sugarcane is an economically important tropic crop primarily for sugar production but increasingly for biofeul production. Its large polyploid genome coupled with interspecific hybridization and aneuploid hindered sugarcane genomic research. The available genome sequence of sorghum, a closely relative of sugarcane, provides an exceptional opportunity to unravel the complex sugarcane genome. In this study, we strategically selected 20 sugarcane BACs each corresponding to a sorghum chromosome arm for sequencing to study the genome structure and organization. Sequence comparisons revealed that sugarcane genome is mostly collinear in the genic regions with sorghum genome, and the coding region of sugarcane and sorghum shared an average of 95.2% sequence identity. The unaligned regions between sugarcane and sorghum sequences were occupied mostly by repeats. The sorghum genome is an excellent template for assembling the genic DNA of the autopolyploid sugarcane genome. The comparable gene density between sugarcane and sorghum and the high number of sugarcane specific genes indicated that sugarcane genome might have retained more, not less, genes after the divergence of these two genera. Polyploidy species might not have faster pace of gene loss despite the redundancy of multiple alleles at each locus.

## Methods

### Selection of sugarcane BACs and sequencing

A sugarcane BAC library constructed from a commercial cultivar R570 was used for physical mapping of the sugarcane genome along with the sorghum genome by overgo hybridization [17,26].

The detailed procedures for cloned, large insert genomic DNA isolation entailed a modified cleared lysate procedure as described in detail earlier [34,35]. BAC DNA was prepared for sequencing on the 454/Roche GS-FLX as described by the manufacturer [36]. Briefly this entailed shearing the purified BAC DNA via nebulization and subsequent end repair, as described [37], followed by ligation of adapter sequences and a second round of end repair to yield a blunt ended DNA library that then was quantified and pooled into 5 horizontal and 4 vertical pools (9 pools for 20 BACs total) using the Clone-Array Pooled Shotgun Sequencing strategy [38-40]. After dilution and emPCR amplification [36], the DNAs were loaded onto a 454/Roche GE-FLX for massively parallel pyrosequencing. The resulting sequence data was deconvoluted to individual BAC shotgun reads that then were assembled, first using the manufacturer supplied Newbler assembler and then by assembly with Phrap [41].

The GenBank accession numbers of these 19 BACs are: FJ348715-FJ348733. One of the 20 BACs has no insert.

### BAC Sequence Annotation

The assembled BAC contig sequences were aligned to each other to identify and exclude ambiguous sequences such as sequence duplication, overlapping, and imbedding. The repeat sequences were masked in the un-ambiguous assembled sequence by RepeatMasker using a known repeats database combined from RepBase databases, TIGR Plant Repeat Databases. Genes were firstly annotated based on the spliced alignment of unmasked sequence assembly to sugarcane expression sequence tag (EST) from TIGR Plant Transcript Assemblies (*Saccharum officinarum *release 2). The reference gene set was further enriched by alignment of the unmasked assembly to closely related sorghum orthologues identified from sorghum EST and annotated CDs.

### Sanger sequencing

Two BAC clones, SC172L01 (60 kb) and SC11815 (80 kb), were sequenced using the shotgun approach with at least 10× coverage using Sanger sequencers. Approximately 3 ug BAC DNA was randomly sheared by a nebulizer (Invitrogen Corp.Carlsbad, CA USA) to produce fragments of 2-4 kb and then precipitated with Pellet Paint Co-Precipitant (Novagen, Darmstadt, Germany). The blunt-ends of DNA fragments were repaired using DNA terminator end repair kit (Lucigen, WI 53562 USA). The fragments with approximately 3 kb size were selected by cutting the gel slide, which was separated on a 1% agarose gel in 1 × TAE buffer through electrophoresis and purified using QIAquick Gel Extraction kit (Qiagen, Germantown, MD). The ligation and transformation were conducted by using Cells Clone Smart Blunt Cloning Kits (Lucigen, WI 53562 USA) following the manufacturer's instruction. DNAs from the 3-kb libraries were isolated through high through-put plasmid DNA miniprep and then subjected to cycle-sequence with ABI BigDye Terminator v3.1 and analyzed on a 3730XL DNA Analyzer (Applied Biosystems, Foster City, CA, USA). These two BACs were sequenced with 10× coverage.

Phred/Phrap/Consed and CAP3 packages were used for sequence assembly. Gaps in assembly and regions of low-quality were resolved by resequencing subclones identified by Autofinish, sequencing PCR products, and/or additional random subclone sequencing. All BAC clones were manually examined for signs of mis-assembly. Suspect regions were clarified either by ambiguous read removal, PCR amplification and sequencing, and/or alignment with a neighboring BAC. A BAC was not considered complete until all inconsistent read pairs were resolved and Consed reported an error rate of less than 1/10,000 bases. The GenBank accession numbers of the two BAC are: GU207345 and GU207346.

### Comparative analysis of sugarcane and sorghum sequence

To identify the corresponding sorghum super contigs of the sugarcane BAC sequences, genes on sugarcane were aligned to the sorghum genome sequence in the web based BLAST search engine, http://www.phytozome.net/search.php?show=blast. The corresponding sorghum super contigs were used as anchor sequences to arrange the order (flip if necessary) of the sugarcane BAC sequence assemblies. The pairwise sequence comparisons and alignments between the arrayed sugarcane assembly and corresponding sorghum sequence were carried out on a visualized sequence alignment program WebACT [42]. The sequence collinearity and local rearrangements were identified based on the above alignments with a bit value > 200.

### Divergence time estimation between species

Orthologous gene pairs were identified. Protein sequences of orthologous gene pairs were aligned by CLUSTALW [43] and the protein alignments were converted back to DNA alignments using PAL2NAL [44]. A few alignments were not reliable and discarded from further analysis. Ks (synonymous substitutions per synonymous site) values for these gene pairs were calculated using Nei-Gojobori method [45] implemented in PAML [46] package. The median Ks value was taken. The species divergence time were estimated by this formula: T_div = Ks/(2*6.5e^-9^). We used the commonly accepted synonymous substitution rate for grass lineage, estimated by Gaut et al. [47].

### Closing gaps between BAC contigs

The gap sizes between the ordered adjacent contigs of each sugarcane BACs were estimated based on the corresponding sorghum gapless sequence. For the gap sizes less than 2 kb, primers were designed from two borders of the flanked contigs to amplify the fragment for gap filling by sequencing the PCR products. Primers were synthesized by Bioneer Inc (Alameda, CA 94501).

The PCR condition were as following: 10- μl PCR mix contained 1-ul of glycerol BAC stock as template DNA, 1× PCR buffer, 0.15 mM of each dNTP, 2.0 mM MgCl_2_, 0.15 μM of reverse and forward primers, and 0.8 units of *Taq *polymerase. The PCR reactions were performed using a 2720 Thermal Cycler (Applied Biosystems, USA), in which the reaction mixture was incubated at 94°C for 6 min, then for 35 cycles of 30 s of denaturing at 94°C, 40 s of annealing at 55°C, and 55 s of extension at 72°C, and then with a final extension at 72°C for 7 min. PCR products were separated on 1.5% agarose gels. The PCR products with single band were purified by using QIAquick PCR purification kit (QIAGEN Science, Maryland, USA) and sequenced at an ABI 3730XL core facility at Biotechnology Center of University of Illinois at Urbana- Champaign.

### RT-PCR

At least one intron was covered by primers designed for RT-PCR experiments to control genomic DNA contamination. Total RNA was extracted from two different tissues, mature leaf and leaf roll, and two genotypes, LA Purple and 95-4655. Approximately 2 μg of total RNA was treated with RNase-free DNase I (Promega, WI, USA) and reverse transcribed using RETROscript kit (Invitrogen, CA, USA). The synthesized cDNAs served as templates for RT-PCR. Four cDNA samples along with BAC DNA as positive control, RNA mix without DNase treatment and RNA mix after DNase treatment, were used as templates for PCR amplification. PCR products were run in 1% agarose gel.

## Authors' contributions

RM, AHP, JB, and BR conceived the study and designed the experiments. SM, FN, and JW carried out 454 Flex sequencing and BAC sequence assembly. JW constructed the shotgun libraries for two BACs and QY sequenced and assembled the BACs using Sanger sequencers. JEM tested insert sizes and CC carried out RT-PCR experiments. JW, HB, JB, CC, MAVS, DSR, MEH, SPM participated in data analysis and interpretation. RM, JW, BR, and AHP drafted and revised the manuscript. All the authors read and approved the manuscript.

## Supplementary Material

Additional file 1 Selection of 20 sugarcane BACs using overgo hybridization. Click here for file

Additional file 2 Row and column pools of sugarcane BACs for 454 sequencing. Click here for file

Additional file 3 Gene and repeat distribution on sugarcane BACs. Click here for file

Additional file 4 RT-PCR confirmation of 26 sugarcane genes inferred by sorghum ESTs. Click here for file

Additional file 5 RT-PCR confirmation of 21 sugarcane genes predicted by sorghum gene models. Click here for file

Additional file 6 List of 44 SSR markers developed from the sugarcane BAC sequences. Click here for file

Additional file 7 **Repetitive sequences in the expanded region of the sorghum genome**. Alignment between sugarcane and sorghum homologous sequences showed DNA sequence expansion in the sorghum genome. The expanded DNA sequences are mostly transposable elements. Click here for file

Additional file 8 **Tandem duplication of putative genes in sugarcane genome.** These genes were identified by aligning the genome sequences with sorghum annotated CDs. Gene B has two copies in sugarcane and one in sorghum. The putative function of genes A, B, C, D, and E are serine carboxypeptidase 2, receptor kinase, OSH15 protein, and homeobox transcription factor GNARLY1, respectively. Click here for file

Additional file 9 **Dot plot alignments between the 454 FLEX assembly, Sanger assembly and the corresponding sorghum regions for BAC SC118L15 and SC172L01**. In both cases, the more complete assembly was put on the x-axis to represent the sugarcane BAC (FLEX assembly for SC118L15, Sanger assembly forSC172L01). The dot plot was based on a word size of 10, i.e. each dotrepresent a 10-mer hit. Green bands were used to visually separate individual contigs. Corresponding sorghum regions were identified as chromosome number: base range in megabase unit. Click here for file

Additional file 10 Summary of repetitive sequences in sugarcane BACs, SC118L15 and SC172L01, and the orthologous euchromatic regions of sorghum. Click here for file
