# Supplementary material for: Microcollinearity between autopolyploid sugarcane and diploid sorghum genomes
Source: BMC Genomics. 2010 Apr 23;11:261. doi: 10.1186/1471-2164-11-261 (PMC2882929; doi:10.1186/1471-2164-11-261)
Supplement: Additional file 7 — Repetitive sequences in the expanded region of the sorghum genome. Alignment between sugarcane and sorghum homologous sequences showed DNA sequence expansion in the sorghum genome. The expanded DNA sequences are mostly transposable elements. [file 1471-2164-11-261-S7.DOC]

Additional File 7. Summary of repetitive sequences in sugarcane BACs, SC118L15 and SC172L01, and the homologous euchromatic regions of sorghum.

| Repeat elements | **2 Sugarcane** BAC sequences | | **sorghum** homologous region | |
| --- | --- | --- | --- | --- |
|  | Length (bp) | Percentage of the sequence (%) | Length (bp) | Percentage of the sequence (%) |
| **Retroelements** | 23897 | **32.02** | 10894 | **13.97** |
| SINEs: | 0 | 0 | 0 | 0 |
| LINEs: | 3721 | 4.65 | 0 | 0 |
| RTE/Bov-B | 3365 | 4.1 | 0 | 0 |
| L1/CIN4 | 356 | 0.55 | 0 | 0 |
| LTR elements: | 20176 | 27.37 | 10894 | 13.97 |
| Ty1/Copia | 10014 | 14.11 | 8140 | 10.44 |
| Gypsy/DIRS1 | 9879 | 12.81 | 2754 | 3.53 |
| **DNA transposons** | 6412 | **8.54** | 4528 | **9.04** |
| hobo-Activator | 367 | 0.49 | 970 | 2.5 |
| Tc1-IS630-Pogo | 770 | 1.1 | 872 | 1.45 |
| En-Spm | 2684 | 3.31 | 254 | 0.33 |
| MuDR-IS905 | 786 | 0.96 | 0 | 0 |
| Tourist/Harbinger | 1644 | 2.42 | 2140 | 3.86 |
| Unclassified: | 0 | 0 | 0 | 0 |
| Simple repeats: | 170 | 0.24 | 1412 | 3.35 |
| Low complexity: | 725 | 1.01 | 1206 | 2.63 |
| **Total** | 31204 | **41.81** | 18040 | **28.99** |
