# Supplementary material for: Microcollinearity between autopolyploid sugarcane and diploid sorghum genomes
Source: BMC Genomics. 2010 Apr 23;11:261. doi: 10.1186/1471-2164-11-261 (PMC2882929; doi:10.1186/1471-2164-11-261)
Supplement: Additional file 8 — Tandem duplication of putative genes in sugarcane genome. These genes were identified by aligning the genome sequences with sorghum annotated CDs. Gene B has two copies in sugarcane and one in sorghum. The putative function of genes A, B, C, D, and E are serine carboxypeptidase 2, receptor kinase, OSH15 protein, and homeobox transcription factor GNARLY1, respectively. [file 1471-2164-11-261-S8.DOC]

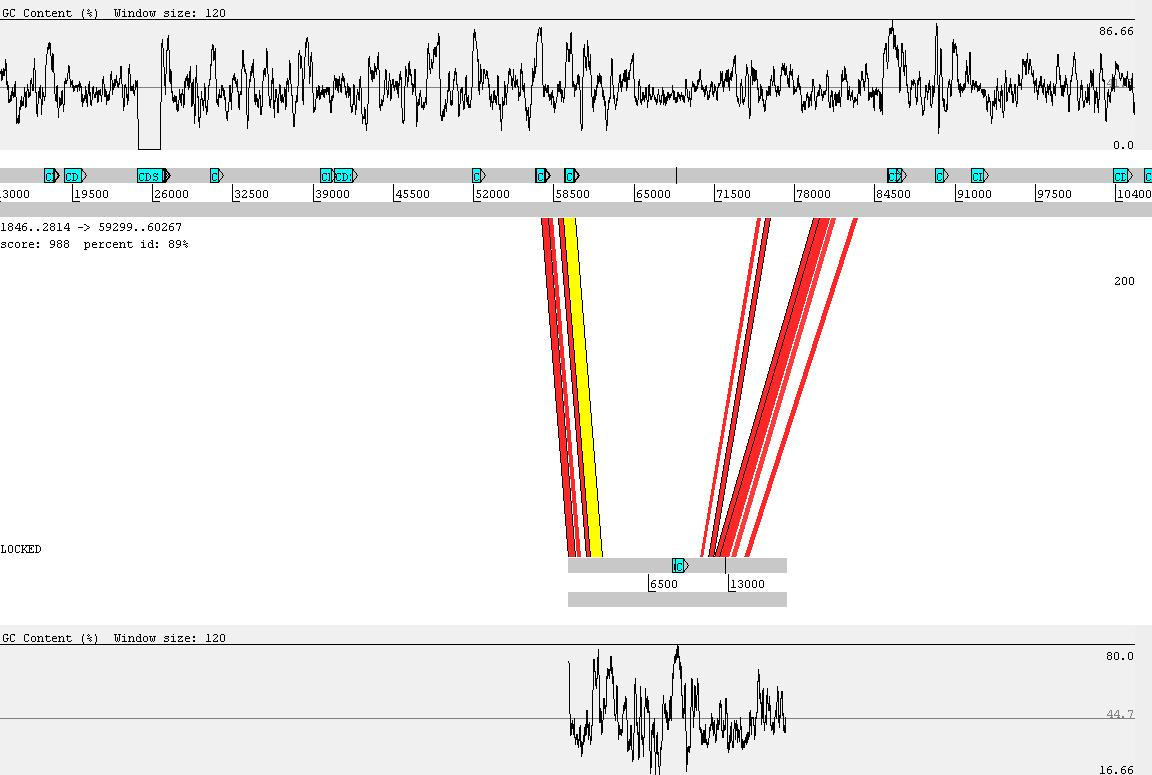


Sorghum chromosome 1 (partial)

Sugarcane 79A20 contig 10

No gene, but 28 LTR retroelements and couple of transposons

No gene, but 10 repeat elements including 4 transposons

Additional File 8
