# Supplementary figures and images for: Microcollinearity between autopolyploid sugarcane and diploid sorghum genomes
Source: BMC Genomics. 2010 Apr 23;11:261. doi: 10.1186/1471-2164-11-261 (PMC2882929; doi:10.1186/1471-2164-11-261)

## Slide 1
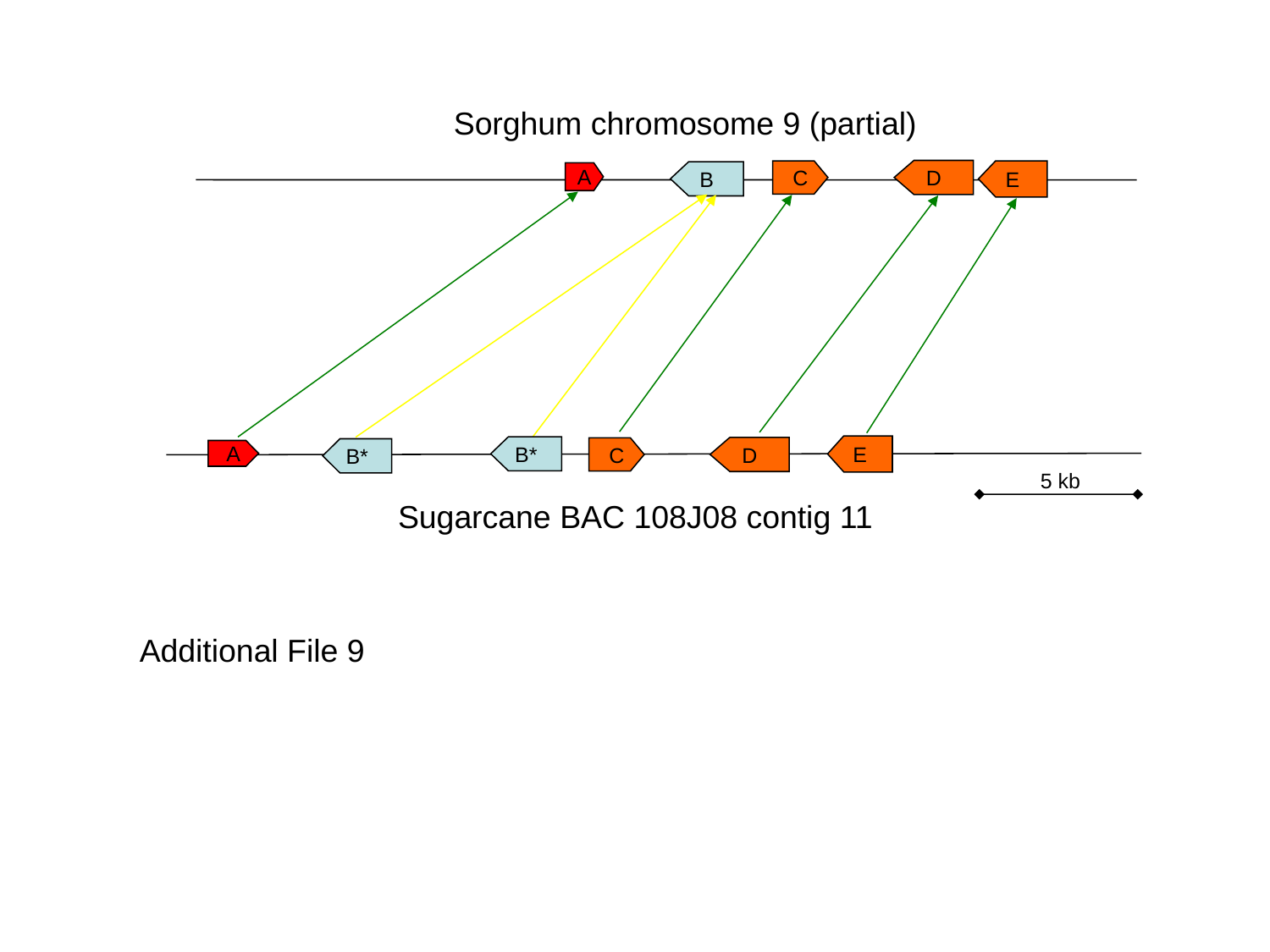

Sorghum chromosome 9 (partial)
D
C
E
B
A
E
B*
D
C
B*
A
5 kb
Sugarcane BAC 108J08 contig 11
Additional File 9

Supplement: Additional file 9 — Dot plot alignments between the 454 FLEX assembly, Sanger assembly and the corresponding sorghum regions for BAC SC118L15 and SC172L01. In both cases, the more complete assembly was put on the x-axis to represent the sugarcane BAC (FLEX assembly for SC118L15, Sanger assembly forSC172L01). The dot plot was based on a word size of 10, i.e. each dotrepresent a 10-mer hit. Green bands were used to visually separate individual contigs. Corresponding sorghum regions were identified as chromosome number: base range in megabase unit. [file 1471-2164-11-261-S9.PPT]
