# Supplementary material for: Microcollinearity between autopolyploid sugarcane and diploid sorghum genomes
Source: BMC Genomics. 2010 Apr 23;11:261. doi: 10.1186/1471-2164-11-261 (PMC2882929; doi:10.1186/1471-2164-11-261)
Supplement: Additional file 10 — Summary of repetitive sequences in sugarcane BACs, SC118L15 and SC172L01, and the orthologous euchromatic regions of sorghum. [file 1471-2164-11-261-S10.PDF]

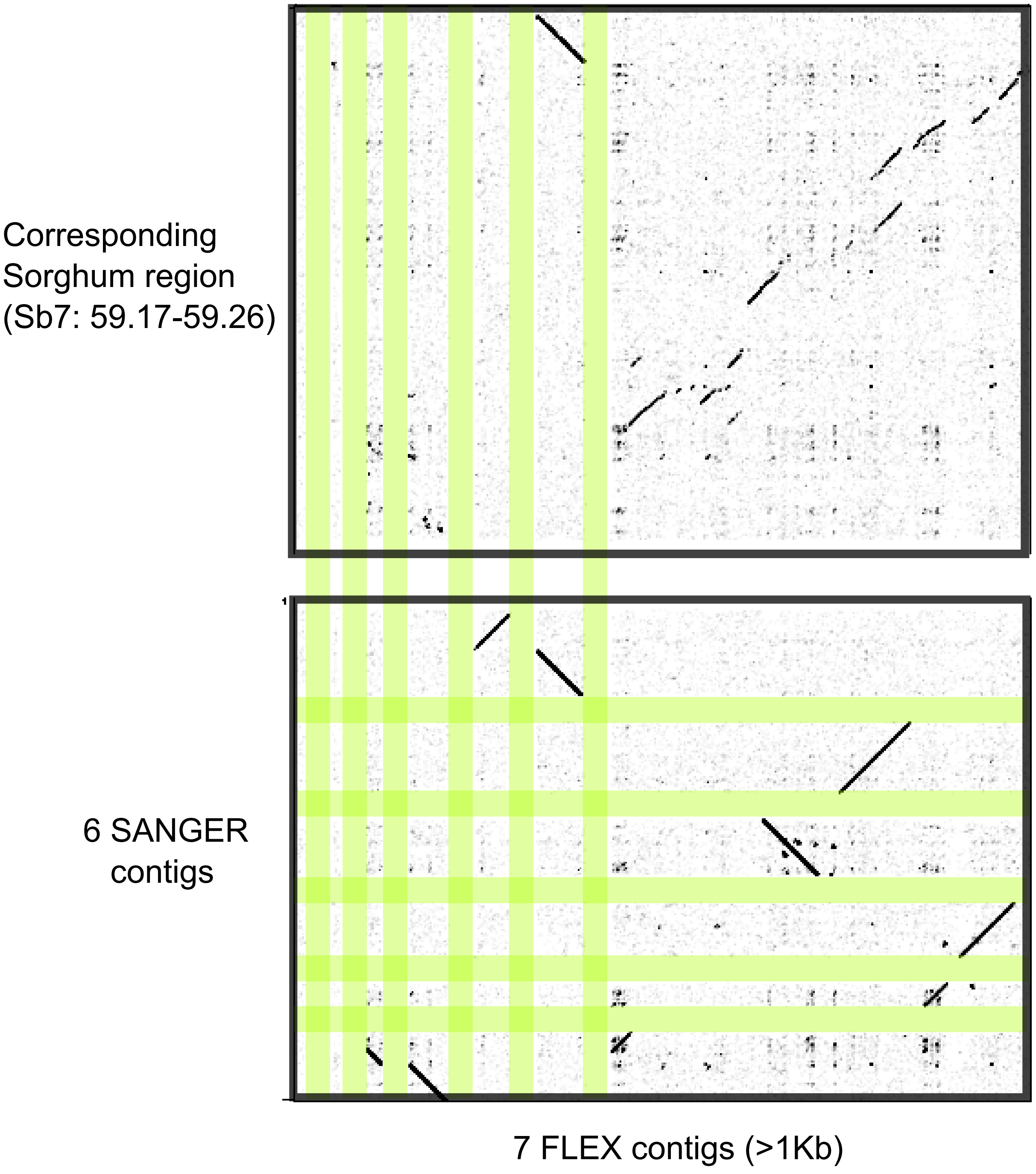

BAC #11 (SC118L15)

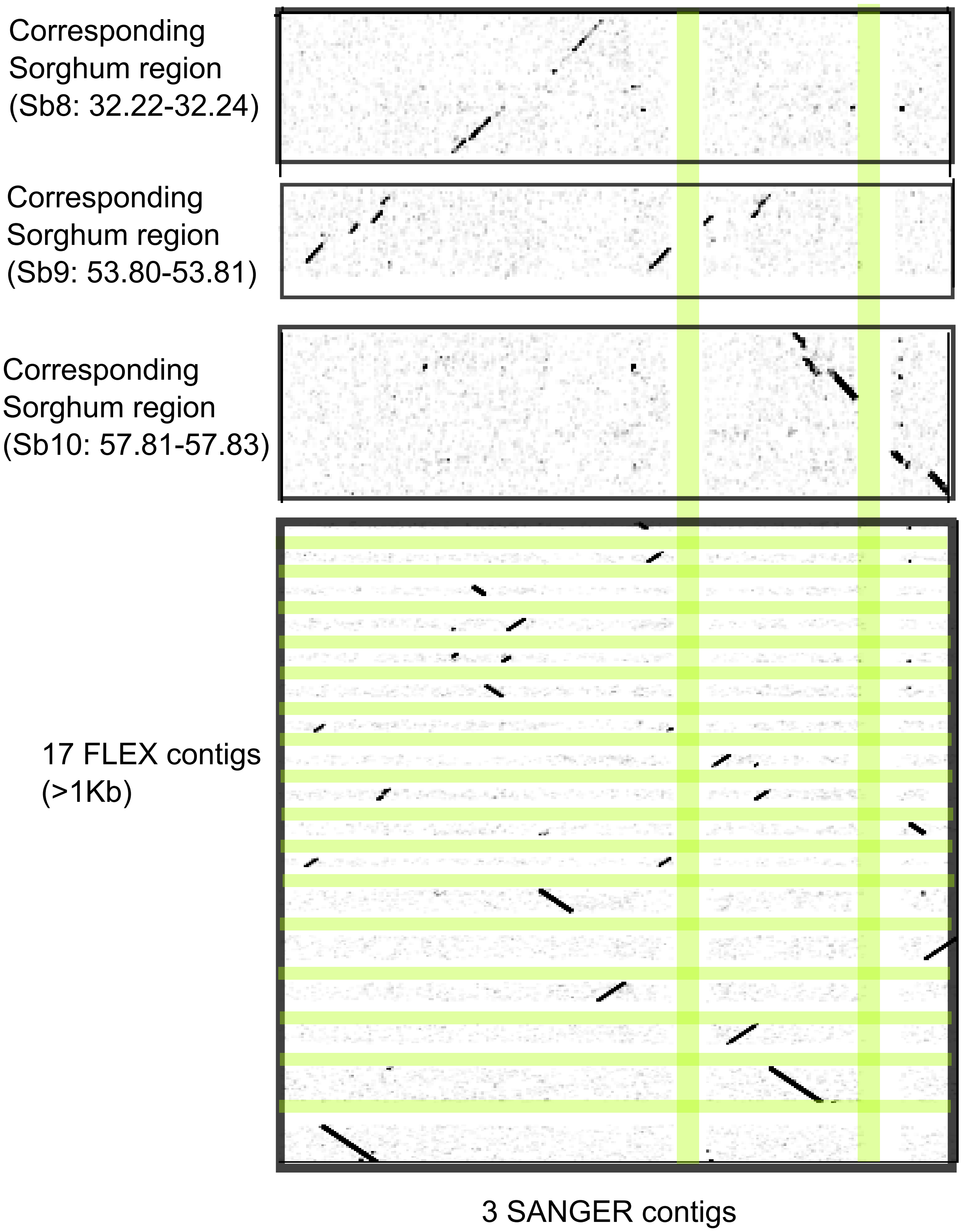

BAC #16 (SC172L01)
